# Vitamin D receptor is associated with prognostic characteristics of breast cancer after neoadjuvant chemotherapy—an observational study

**DOI:** 10.3389/fonc.2024.1458124

**Published:** 2024-10-01

**Authors:** Joanna Streb, Agnieszka Łazarczyk, Przemysław Hałubiec, Anna Streb-Smoleń, Julita Ciuruś, Magdalena Ulatowska-Białas, Martyna Trzeszcz, Kamil Konopka, Diana Hodorowicz-Zaniewska, Joanna Szpor

**Affiliations:** ^1^ Department of Oncology, Jagiellonian University Medical College, Cracow, Poland; ^2^ University Center of Breast Disease, University Hospital, Cracow, Poland; ^3^ Department of Pathomorphology, Jagiellonian University Medical College, Cracow, Poland; ^4^ Department of Pathomorphology, University Hospital, Cracow, Poland; ^5^ Doctoral School of Medical and Health Sciences, Jagiellonian University Medical College, Cracow, Poland; ^6^ Department of Oncology, Maria Sklodowska-Curie National Research Institute of Oncology, Cracow, Poland; ^7^ Corfamed Woman’s Health Center, Wroclaw, Poland; ^8^ Department of Pathology and Clinical Cytology, University Hospital in Wroclaw, Wroclaw, Poland; ^9^ General, Oncological, and Gastrointestinal Surgery, Jagiellonian University Medical College, Cracow, Poland; ^10^ Breat Unit, Department of General Surgery, University Hospital, Cracow, Poland

**Keywords:** breast cancer, neoadjuvant chemotherapy, vitamin D receptor, vitamin D, residual cancer burden, pathological response

## Abstract

**Background:**

Breast cancer (BC) is the most commonly diagnosed malignant tumor in women. The disease and its subsequent treatment pose a serious burden on the quality of life of patients. Neoadjuvant chemotherapy (NAC) has become one of the crucial strategies for the management of BC. Since the identification of the vitamin D receptor (VDR) in mammary tissues, extensive mechanistic research has been conducted on its function. The expression of VDR in BC cells and the tumor microenvironment could be a new prognostic factor for BC after NAC.

**Patients and Methods:**

This observational, single-center study compared data from clinical and histopathological records of 111 female subjects with the expression of VDR in different cellular and tissue components of breast specimens obtained from surgery after NAC. VDR expression was evaluated using an immunoreactive score assigned after immunohistochemistry. Intergroup comparisons and logistic regression were used to identify associations between VDR expression and clinicopathological features of BC.

**Results:**

We found that the expression of VDR is associated with various clinical features (i.e., age, menopausal status, and NAC cycle number) and characteristics of prognostic significance, such as residual cancer burden class. Logistic regression analysis revealed that the expression of VDR in the nuclei and cytoplasm of surrounding normal mammary cells predicted vascular invasion and lymph node involvement.

**Conclusions:**

The expression of VDR in tumor cells and their microenvironment is related to the clinicopathological characteristics of BC after NAC.

## 1 Introduction

Breast cancer (BC) is the most commonly diagnosed malignant tumor in women (except for non-melanoma skin cancers), with more than 2.2 million new cases in 2020 according to GLOBOCAN data (1). The disease and subsequent treatment pose a serious burden on the patient’s quality of life, with an estimated loss of disability-adjusted life years exceeding 20 million (2). In countries with high standards of medical care, localized disease has a 5-year relative survival of 99%, while for regionally advanced disease it is approximately 85% (3).

Various well-established risk factors for BC have been identified, both nonmodifiable and modifiable. Examples of the first group include female sex, older age, menopausal status, and genetics (family history and identified gene mutations, i.e., BRCA1, BRCA2, PALB2, CHEK2 and ATM), while the latter includes low level of physical activity, overweight and obesity, alcohol consumption, smoking, and nutritional habits (particularly low vitamin intake) (4, 5).

After confirmation of BC diagnosis, a set of prognostic and predictive factors is routinely assessed to guide further management. Prognostic factors provide information on the general prognosis and anticipated course of breast malignancy, while predictive factors enable us to decide whether additional therapeutic interventions might be beneficial to the patient (6). Generally, most characteristics such as histologic type, nuclear grade, lymph node status, lymphovascular invasion, expression of the progesterone receptor (PR), and proliferation (Ki-67 expression) are considered prognostic, whereas others, that is, expression of the estrogen receptor (ER) or human epidermal growth factor receptor 2 (HER2) status, are used for both purposes.

Neoadjuvant chemotherapy (NAC) has become a crucial strategy for the management of BC, and the current paradigm is that it should be considered in the majority of cases that would otherwise require adjuvant chemotherapy after breast surgery (7). NAC would ideally lead to a reduction in tumor size and degree of lymph node involvement, which is referred to as downstaging, allowing less extended surgery (ideally breast-conserving instead of mastectomy). Pathological complete response (pCR) is usually recognized as a satisfactory predictor of event-free and overall survival in patients with BC (8). Some authors have suggested the use of NAC even in early stage BC to improve cosmetic effects after surgery and to reduce the risk of complications afterwards (i.e., lymphoedema) (9). Currently, the main obstacle for NAC in BC is the increased frequency of local recurrence compared to adjuvant therapy (relative risk [RR] = 1.37, 95%CI 1.17-1.62), as established by the Early Breast Cancer Trialists’ Collaborative Group (10).

The chemotherapeutics used in NAC are anthracyclines, taxanes, and cyclophosphamide, although platinum(IV) derivatives are administered in some cases (11). In HER2-positive tumors, a targeted anti-HER2 drug should be added to the NAC regimen.

As mentioned earlier, pCR may be considered a reasonable surrogate endpoint in studies that assess the effectiveness of NAC. Residual cancer burden (RCB) is another metric designed to describe the extent of BC that remains in the original tumor bed and axillary lymph nodes after NAC (12). Class 0 RCB is essentially identical to pCR. It seems reasonable to search for biological features of BC that could be associated with RCB (or its components, i.e., lymph node involvement), as they might be suitable candidates for updated predictive models or even as targets for novel therapeutic interventions.

Vitamin D receptor (VDR) was first identified in BC cells in 1979 (13). Since then, extensive mechanistic research has been conducted on its function in the nuclei and cytoplasm (14). The role of VDR in the regulation of breast cell growth and immunoregulation is well known. It was also revealed that high total expression of VDR in BC tissue is associated with better overall patient survival (15).

However, previous analyses have omitted the importance of VDR expression in BC tissues after NAC, leaving a gap in the state of knowledge. Promising results of studies investigating serum 25-hydroxycholecalciferol in patients with BC after NAC justify the requirement for such research (16, 17). The tumor microenvironment (immune cell infiltration, surrounding breast lobules, and ducts) should also be considered because the expression of VDR in these compartments could also play a role.

Therefore, the objective of this study was to evaluate the relationships between the expression of VDR and basic clinicopathological characteristics together with those of a well-established prognostic role in tissue material received from the postoperative sample of BC after NAC.

## 2 Patients and methods

### 2.1 Study subjects

The data for this observational, single-center study were derived from the clinical and pathology reports of patients diagnosed in the Department of Pathomorphology of the University Hospital in Cracow (Poland) between 2015 and 2021.

The analyzed samples were collected from January 2016 to December 2020. The hospital database was screened for patients who had a confirmed diagnosis of BC within a given time range, and the following criteria were used to qualify them for the study: female sex; histological diagnosis of BC from core needle biopsy; NAC treatment (no hormone therapy) and subsequent breast surgery; no malignancy other than BC diagnosed.

If the subject met the above requirements, her clinical and pathological data were collected from hospital records. These included demographic characteristics, information about surgery, NAC regimen, number of cycles, and routinely acquired histological data.

Study flow-chart is available as **Supplementary Figure S1**.

All patients underwent a core needle biopsy, and a postoperative samples were evaluated by a pathologist experienced in BC diagnosis. The clinical stage before neoadjuvant treatment and pathological stage after surgery were determined according to the eighth edition of the AJCC guidelines from 2017 (18). Grading was performed using the Nottingham Histologic Grade system. The score and RCB class were evaluated according to the Residual Cancer Burden Calculator (19).

Oncological treatment was governed by a specialist from the Oncology Clinical Department of the University Hospital in Cracow, and surgery was performed in the Breast Unit of the University Hospital in Cracow. All therapeutic procedures followed the ESMO guidelines (20).

Histological slides of routinely processed, formalin-fixed, and paraffin-embedded tissue specimens of postoperative BC material were retrieved from the archive and immunostained, as described in the following section.

This study adhered to all principles of the Declaration of Helsinki of 1975 (revised in 2013), and its protocol was approved by the Bioethical Committee of Jagiellonian University (protocol code 1072.6120.289.2020 and date of approval: October 28, 2020).

The consent was informed and obtained written.

### 2.2 Immunohistochemistry

Immunohistochemical visualization of all antigens was performed following routine procedures in our laboratory (**Supplementary Table S1**). ER, PR, and HER2/neu were immunostained using a BenchMark Ultra immunostainer (Roche Ventana, Tucson, AZ, USA) and Ki-67 on a DAKO Omnis immunostainer (Dako, Santa Clara, CA, USA). All stains were performed automatically. Control breast tissue was used for ER and PR, while BC tissue was used for HER2 (both positive and negative). For Ki-67, few control tissues (appendix, liver, pancreas, and tonsils) were used.

Both ER and PR expressions were considered positive if ≥1% of BC cells had positively stained nuclei. High Ki-67 expression was observed if ≥20% of tumor cells were positive. HER2 status was established according to the practice guidelines of the American Society of Clinical Oncology and the American College of Pathologists (21). For cases with a HER2 score of 2+, additional FISH was performed using a ZytoLight FISH-Tissue Implementation Kit (ZYTOVISION GmbH, Bremerhaven, Germany) according to the manufacturer’s protocol. Nucleic acid denaturation and hybridization were performed using the CytoHYB CT500 automatic system (CytoTest Inc., Rockville, MD, USA). Finally, the locus-specific identifier signals from the HER2/neu end of CEP17 were counted using a fluorescence microscope and compared. If the HER2/neu to CEP17 ratio was greater than 2.0, overexpression was considered (21).

### 2.3 Assessment of VDR immunoreactive score

After immunostaining, each slide was scanned using an Aperio GT 450 DX scanner (Leica Biosystems). Visualization and analysis of virtual slides were conducted using MedLan Slide Viewer software v.1.11 (MedLan, Poland).

**Figure 1:**
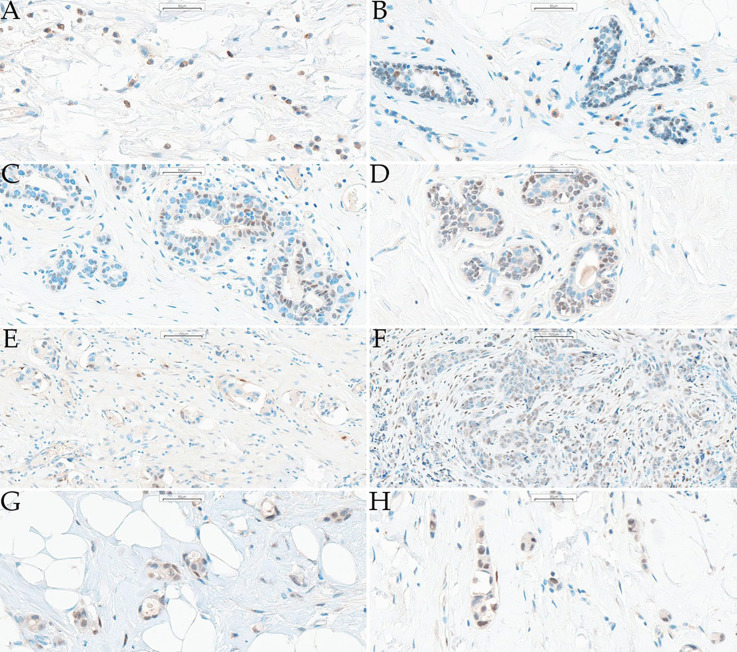
Examples of analyzed slides together with assigned scores. **(A)** Magn. 400x, immune infiltrate, VDR-IRS – percentage of stained cells: 4 pts, intensity: 2 pts; **(B)** Magn. 400x, normal lobules, nuclear VDR-IRS – percentage of stained cells: 2 pts, intensity: 1 pt; cytoplasmic VDR-IRS – 0 pts; immune infiltrate VDR-IRS – percentage of stained cells: 2 pts, intensity: 2 pts; **(C)** Magn. 400x, normal lobules, nuclear VDR-IRS – percentage of stained cells: 2 pts, intensity: 2 pts; cytoplasmic VDR-IRS – percentage of stained cells: 3 pts, intensity: 1 pts; **(D)** Magn. 400x, normal lobules, nuclear VDR-IRS – percentage of stained cells: 3 pts, intensity: 3 pts; cytoplasmic VDR-IRS – percentage of stained cells: 4 pts, intensity: 1 pt; **(E)** Magn. 200x, cancerous foci, nuclear VDR-IRS – percentage of stained cells: 1 pt, intensity: 1 pt; cytoplasmic VDR-IRS – percentage of stained cells: 4 pts, intensity: 1 pt; **(F)** Magn. 200x, cancerous foci, nuclear VDR-IRS – percentage of stained cells: 4 pts, intensity: 1 pt; cytoplasmic VDR-IRS – percentage of stained cells: 4 pts, intensity: 1 pt; **(G)** Magn. 400x, cancerous foci, nuclear VDR-IRS – percentage of stained cells: 4 pts, intensity: 2 pts; cytoplasmic VDR-IRS – cytoplasmic: 4 pts, intensity: 2 pts; **(H)** Magn. 400x, cancerous foci, nuclear VDR-IRS – percentage of stained cells: 4 pts, intensity: 3 pts; cytoplasmic VDR-IRS – percentage of stained cells: 4 pts, intensity: 2 pts. magn, magnification; VDR-IRS, vitamin D receptor immunoreactive score; pt(s)–point(s).

The vitamin D receptor immunoreactive score (VDR-IRS) was evaluated at low magnification (×200) by a senior pathologist and pathology resident following the standard approach proposed by Remmele and Stegner and was used for VDR successfully (22, 23). VDR-IRS was calculated separately for the cytoplasm and nuclei of cells in normal lobules and cancerous foci (where possible, i.e., in cases with RCB>0), whereas for immune cell infiltration, one value of VDR-IRS was established because nuclear and cytoplasmic stains were not clearly distinguishable. The intensity was scored on an ordinal scale from 0 to 3 points (pts) (A), while the proportion of positively stained cells was classified into five ranges: 0%–0 pts,<10%–1 pt, 10-50% - 2 pts, 51-80% - 3 pts, and >80%–4 pts (B). Each score was assigned for each slide by the aforementioned assessors through discussion, and the final IRS was calculated as product A × B (range 0-12 pts). Examples of the scores assigned to different samples are presented in **Figure 1**.

### 2.4 Statistical analysis

All nominal data were presented as absolute frequencies (N) and proportions (%), while interval data are shown as mean ± standard deviation or median (interquartile range) depending on the type of data (continuous or interval) and distribution. The normality of continuous data was determined by visual assessment of histograms and the Shapiro-Wilk test. Because the main variable of interest for the majority of comparisons was ordinal (VDR-IRS), non-parametric tests were used for further analysis.

Analyses were carried out for the whole group of subjects and for the subgroup selected with the RCB>0 criterion (for comparisons with VDR-IRS of cancerous foci). Four additional variables were derived from the initial VDR-IRS data:


$$\Delta_{\text{nuclear}}\text{ VDR-IRS }=\;\left(\text{cancerous foci nuclear VDR}-\text{IRS}\right)\;–\left(\text{normal lobules nuclear VDR}-\text{IRS}\right)$$

$$\Delta_{\text{cytoplasmic}}\text{ VDR}-\text{IRS}=\left(\text{cancerous foci cytoplasmic VDR}-\text{IRS}\right)–\left(\text{normal lobules cytoplasmic VDR}-\text{IRS}\right)$$

$$\text{normal lobules }\Delta_{\text{n}-\text{c}}\text{ VDR}-\text{IRS}=\left(\text{normal lobules nuclear VDR}-\text{IRS}\right)\;–\left(\text{normal lobules cytoplasmic VDR}-\text{IRS}\right)$$

$$\text{cancerous foci }\Delta_{\text{n}-\text{c}}\text{ VDR}-\text{IRS}=\left(\text{cancerous foci nuclear VDR}-\text{IRS}\right)–\left(\text{cancerous foci cytoplasmic VDR}-\text{IRS}\right)$$

The initial rationale for Δ_nuclear_ and Δ_cytoplasmic_ VDR-IRS was to create a variable that would adjust the VDR-IRS observed in cancerous tissue to the VDR-IRS of normal lobules, hypothetically reducing the confounding effect of external factors (such as differences in vitamin D synthesis and intake or its concentration in the subjects’ serum). The purpose of Δ_n-c_ VDR-IRS was to add a variable that accounts for the difference in the pool of nuclear and cytoplasmic VDR, considering that the potential shifts of VDR in the cellular compartments are important for the biological effects of VDR.

Spearman’s rank correlation coefficient R was used to describe the relationships between the interval variables. For comparison between two groups, the Mann-Whitney U test was used, while differences between more than two groups were assessed using Kruskal-Wallis ANOVA with *post-hoc* Dunn’s test. Multivariate stepwise logistic regression models with 10-fold cross-validation were constructed, and the most promising models were selected according to the highest Nagelkerke pseudo-R^2^ values and best fit in the Hosmer–Lemeshow test.

Furthermore, we divided the VDR-IRS into dichotomous categories with a cutoff value of 8, based on the results of previous analyses (24) and the distribution of scores in our study group. A VDR-IRS score< 8 was defined as a ‘low score’, while a VDR-IRS score ≥ 8 was defined as a ‘high score’. Comparisons between binomial variables were performed using a two-sided Fisher’s exact test.

The frequency of type I errors (i.e., the threshold for significance) was set with α = 0.05. However, to avoid false positive results due to multiple comparisons, a Benjamini-Hochberg correction was applied with the assumption of a false discovery ratio (FDR) equal to 0.05 and p^BH^ values are also reported for comparisons that initially showed p<0.05.

Statistical analysis was performed the Statistica 13.3 software (Statsoft Inc., Tulsa, OK, USA).

### 2.5 Data availability

The data presented in this study are available upon request from the corresponding author. The data are not publicly available because of privacy restrictions.

## 3 Results

A summary of the clinical and pathological characteristics of the study participants is provided in **Table 1**. Detailed data on the NAC regimens are provided in **Supplementary Table S2**. The results of VDR-IRS scoring are shown in **Supplementary Figure S2**. Data are disclosed separately for cases in which an RCB of 0 was not achieved after NAC.

**Table 1:** The main characteristics of the study subjects and the information obtained from the pathology reports.

Characteristic	All cases N = 111	Cases without pathological complete response (RCB class > 0) N = 73
Age upon initial diagnosis (years)	51.0 ± 13.0	52.2 ± 13.1
Positive menopausal status	56 (52%)	40 (56%)
Time from diagnosis to operation (months)	6.3 ± 1.1	6.1 ± 1.2
*Surgery type*		
Breast conserving Mastectomy	57 (51%) 54 (49%)	30 (41%) 43 (59%)
*Lymph node surgery*		
Sentinel lymph node biopsy Axillary lymph nodes dissection	61 (55%) 50 (45%)	27 (37%) 46 (63%)
*Clinical stage (T feature)*		
1 2 3 4	9 (8%) 60 (54%) 26 (23%) 15 (14%)	3 (4%) 37 (51%) 19 (26%) 13 (18%)
*Clinical stage (N feature)*		
0 1 2 3	38 (34%) 60 (54%) 4 (4%) 8 (7%)	20 (28%) 43 (60%) 4 (6%) 5 (7%)
Number of NAC cycles	10.5 ± 4.7	9.8 ± 4.9
*Chemotherapeutics*		
Anthracyclines Cyclophosphamide Platinum (IV) derivatives Taxanes	102 (92%) 98 (88%) 33 (30%) 104 (94%)	66 (90%) 62 (85%) 12 (16%) 66 (90%)
*Immunotherapy*		
Trastuzumab Trastuzumab + pertuzumab	14 (13%) 21 (19%)	4 (5%) 17 (23%)
Data from pathology reports		
TIL (%)	6.0 ± 7.5	6.1 ± 8.4
*Histological type of tumor*		
no-special type invasive lobular carcinoma other	92 (83%) 6 (5%) 13 (12%)	57 (78%) 6 (8%) 10 (14%)
*Molecular subtype of tumor*		
luminal A luminal B HER2- luminal B HER2+ non-luminal HER2+ triple-negative breast cancer	4 (4%) 41 (37%) 24 (22%) 15 (14%) 27 (24%)	4 (5%) 35 (48%) 14 (19%) 9 (12%) 10 (14%)
Ki-67 (%, in core needle-biopsy)	48.8 ± 23.2	42.8 ± 21.1
RCB score	1.83 ± 1.63	2.78 ± 1.19
*RCB class*		
0 I II III	38 (34%) 11 (10%) 36 (32%) 26 (23%)	- 11 (15%) 36 (49%) 26 (36%)
*Pathological response*		
pCR pPR pNR	38 (34%) 61 (55%) 12 (11%)	- 61 (84%) 12 (16%)
*Nuclear grade (before NAC)*		
G1 G2 G3	7 (6%) 49 (44%) 55 (50%)	6 (8%) 39 (53%) 28 (38%)
*Nuclear grade (after NAC)*		
G0 G1	40 (36%) 9 (8%)	2 (3%) 9 (15%)
G2 G3	38 (34%) 13 (12%)	38 (61%) 13 (21%)
*HER2 status*		
positive negative	40 (36%) 70 (63%)	24 (33%) 48 (67%)
Estrogen receptor expression (%)	43.2 ± 41.7	56.0 ± 40.6
Progesterone receptor expression (%)	22.6 ± 31.9	29.1± 34.01
*Pathological stage (T feature)*		
0 DCIS 1 2 3 4	33 (30%) 7 (6%) 42 (38%) 22 (20%) 4 (4%) 3 (3%)	- 2 (3%) 42 (58%) 22 (30%) 4 (5%) 3 (4%)
*Pathological stage (N feature)*		
0 mi 1 2 3	67 (60%) 2 (2%) 19 (17%) 16 (14%) 7 (6%)	29 (40%) 2 (3%) 19 (26%) 16 (22%) 7 (10%)
Vascular invasion (after NAC)	44 (40%)	44 (61%)

^1^data point for one case was missing; ^2^data points for 10 cases were missing. DCIS, ductal carcinoma in situ; HER2, human epidermal growth factor receptor 2; NAC, neoadjuvant chemotherapy; pCR, pathological complete response; pPR, pathological partial response; pNR, pathological non-response; RCB, residual cancer burden; TIL, tumor-infiltrating lymphocytes.

Nominal data are presented as an absolute number (N) and the frequencies whereas the interval data are shown as the mean ± standard deviation.

Correlation analysis of the whole studied sample revealed a moderate negative correlation between Δ_n-c_ VDR-IRS in normal lobules and subjects age (R = -0.31, p^BH^ = 0.014).

In the group with RCB > 0, immune cells infiltrate VDR-IRS were moderately strongly correlated with Ki-67 before NAC (positive, R = 0.34, p^BH^ = 0.036) and ER expression (negative, R = -0.30, p^BH^ = 0.0495). Additionally, Δ_cytoplasmic_ VDR-IRS showed a moderate positive association with the number of NAC cycles (R = 0.41, p^BH^ = 0.005). The results of correlation analysis are presented in **Table 2**; **Supplementary Table S3**.

**Table 2:** Correlations between VDR-IRS and the clinicopathological characteristics of BC.

All cases (RCB class 0-III)										
	normal lobules cytoplasmic VDR-IRS			normal lobules nuclear VDR-IRS		immune cells infiltrate VDR-IRS			normal lobules Δ_n-c_ VDR-IRS	
	Spearman R		p-Value	Spearman R	p-Value	Spearman R	p-Value			
age (years)	0.20		0.047 p^BH^ > 0.05	-0.16	0.1	-0.03	0.7		-0.31	0.0015 p^BH^ = 0.014
Ki-67 before NAC (%)	-0.05		0.6	-0.04	1.0	0.20	0.038 p^BH^ > 0.05		0.05	0.6
RCB	-0.20		0.039 p^BH^ > 0.05	-0.15	0.1	-0.13	0.1		-0.04	0.7
grade after NAC	-0.16		0.1	-0.25	0.016 p^BH^ > 0.05	-0.03	0.8		-0.14	0.2
chemotherapy cycles number	-0.21		0.032 p^BH^ > 0.05	-0.03	0.8	-0.07	0.5		0.16	0.1

Cases without pathological complete response (RCB class > 0)										
	normal lobules cytoplasmic VDR-IRS		normal lobules nuclear VDR-IRS		immune cells infiltrate VDR-IRS		cancerous foci cytoplasmic VDR-IRS		cancerous foci nuclear VDR-IRS	
	Spearman R	p-Value	Spearman R	p-Value	Spearman R	p-Value	Spearman R	p-Value	Spearman R	p-Value
age (years)	0.18	0.2	-0.20	0.1	>0.01	1.0	0.03	0.8	0.14	p0.5
Ki-67 before NAC (%)	-0.05	0.7	-0.04	0.7	0.34	0.004 p^BH^ = 0.036	0.05	0.7	0.13	0.3
RCB	-0.03	0.8	-0.06	0.7	-0.20	0.1	-0.12	0.3	-0.16	0.2
estrogen receptor (%)	0.13	0.3	0.20	0.9	-0.30	0.011 p^BH^ = 0.0495	-0.13	0.3	-0.22	0.1
grade after NAC	0.18	0.2	-0.16	0.2	0.22	0.1	-0.07	0.6	0.12	0.3
chemotherapy cycles number	-0.33	0.008 p^BH^ > 0.05	-0.05	0.7	-0.06	0.6	0.18	0.1	0.25	0.037 p^BH^ > 0.05
	Δ_nuclear_ VDR-IRS			Δ_cytoplasmic_ VDR-IRS		normal lobules Δ_n-c_ VDR-IRS			cancerous foci Δ_n-c_ VDR-IRS	
	Spearman R		p-Value	Spearman R	p-Value	Spearman R	p-Value		Spearman R	p-Value
age (years)	0.33		0.008 p^BH^ > 0.05	-0.07	0.6	-0.32	0.009 p^BH^ > 0.05		0.05	0.7
Ki-67 before NAC (%)	0.14		0.3	0.09	0.5	-0.08	0.5		-0.17	0.1
RCB	-0.17		0.2	-0.10	0.4	-0.01	0.9		0.08	0.5
estrogen receptor (%)	-0.31		0.013 p^BH^ > 0.05	-0.21	0.1	-0.01	1.0		-0.16	0.2
grade after NAC	0.12		0.4	-0.18	0.2	-0.25	0.1		0.10	0.4
chemotherapy cycles number	0.29		0.017 p^BH^ > 0.05	0.41	0.0006 p^BH^ = 0.005	0.20	0.1		0.17	0.1

BC, breast cancer; HER2, human epidermal growth factor receptor 2; NAC, neoadjuvant chemotherapy; RCB, residual cancer burden; TIL, tumor-infiltrating lymphocytes; VDR-IRS, vitamin D receptor-immunoreactive score.

The table presents results separately for all investigated subjects (VDR-IRS of normal lobules and immune cells infiltrate) and for subjects without complete pathological response (VDR-IRS also for cancerous foci). Furthermore, correlations are presented with secondary variables received by subtracting the original VDR-IRS values. Spearman correlation coefficient R was used to represent the direction and magnitude of the relationship. If the p-Value for the coefficient was below 0.05, the Benjamini-Hochberg procedure was applied to verify whether the correlation remains significant after correction for multiple comparisons. The complete results of the correlation analysis are presented in **Supplementary Table S3**.

An investigation of differences in VDR-IRS between groups for all cases revealed that lower cytoplasmic VDR-IRS in normal lobules was associated with a lack of complete pathological response and vascular invasion. In the subgroup with RCB > 0, a positive menopausal status was observed in subjects with higher nuclear VDR-IRS in cancerous foci and vascular invasion among those with lower levels. Consequently, higher Δ _nuclear_ VDR-IRS was observed in women with positive menopausal status (the only comparison between two groups that remained significant after Benjamini-Hochberg correction, p^BH^ = 0.048) and without vascular invasion. Triple-negative BC was more prevalent in subjects with higher values of both Δ _nuclear_ VDR-IRS and Δ_cytoplasmic_ VDR-IRS (i.e., VDR-IRS for both nuclei and cytoplasm was higher in tumor cells than in normal cells).

Comparisons of VDR-IRS among cases without complete pathological response also showed that Δ_cytoplasmic_ VDR-IRS was much higher in subjects with RCB class I (median 4, IQR: 4-5) compared to those with RCB class II (median 0, IQR: 0-3) and RCB class III (median 1, IQR: 0-2) (p^BH^ = 0.01). In the remaining comparisons, the *post hoc* analysis did not show any differences between particular groups, or there were no clear trends.

Comparisons between groups are presented in **Tables 3**, **4** as well as in **Supplementary Tables S4**, **S5**.

**Table 3:** Comparisons of dichotomous groups according to VDR-IRS.

All cases (RCB class 0-III)										
	normal lobules cytoplasmic VDR-IRS		normal lobules nuclear VDR-IRS			immune cells infiltrate VDR-IRS			normal lobules Δ_n-c_ VDR-IRS	
	Median (IQR)	p-Value	Median (IQR)		p-Value	Median (IQR)	p-Value		Median (IQR)	p-Value
*RCB class * 0 I-III	4 (0–4) 3 (0–4)	0.023 p^BH^ > 0.05	6 (4-9) 6 (3-6)		0.1	4 (3-6) 4 (2-6)	0.5		3 (2-5) 3 (1-5)	0.7
*Molecular subtype of tumor * triple-negative breast cancer other	3 (0-4) 2.5 (0-4)	0.5	6 (3-8) 6 (2-6)		0.3	4 (3-6) 4 (2-4)	0.4		3 (1-5) 2 (2-5)	0.7
*ypT * 0 or is 1-4	4 (0-4) 3 (0-4)	0.01 p^BH^ > 0.05	6 (4-9) 6 (3-6)		0.1	4 (2.5-6) 4 (2-4)	0.5		3 (2-5) 3 (1-5)	1.0
*Vascular invasion * absent present	4 (0-4) 2 (0-4)	0.01 p^BH^ > 0.05	6 (3-9) 6 (3-6)		0.4	4 (3-6) 4 (2-6)	0.5		2 (1-5) 3 (2-6)	0.5
*Menopausal status * positive negative	3 (0-4) 2 (0-4)	0.6	6 (3-6) 6 (3-8)		0.3	4 (3-6) 4 (2-6)	0.7		2 (1-5) 3 (2-6)	0.1
Cases without pathological complete response (RCB class > 0)										
	normal lobules cytoplasmic VDR-IRS		normal lobules nuclear VDR-IRS		immune cells infiltrate VDR-IRS		cancerous foci cytoplasmic VDR-IRS		cancerous foci nuclear VDR-IRS	
	Median (IQR)	p-Value	Median (IQR)	p-Value	Median (IQR)	p-Value	Median (IQR)	p-Value	Median (IQR)	p-Value
*Molecular subtype of tumor * triple-negative breast cancer other	0 (0-3) 3 (0-4)	0.2	3 (2-6) 6 (3-6)	0.1	4 (4-4) 4 (2-6)	0.8	4 (4-4) 4 (0-4)	0.03	2.5 (2-8) 2.5 (1-6)	0.6
*Vascular invasion * absent present	3 (0-4) 2 (0-4)	0.1	6 (3-6) 6 (3-6)	0.9	** ** 4 (3-4) 4 (2-6)	0.8	4 (2-4) 4 (0-4)	0.3	4 (2-8) 2 (1-4)	0.031 p^BH^ > 0.05
*Menopausal status * positive negative	3 (0-4) 2 (0-4)	0.8	6 (3-6) 6 (3-8)	0.5	4 (3.5-4) 4 (2-6)	0.4	4 (2.5-4) 3.5 (0-4)	0.1	3.5 (2-7) 2 (1-4)	0.0457 p^BH^ > 0.05
	Δ_nuclear_ VDR-IRS		Δ_cytoplasmic_ VDR-IRS			normal lobules Δ_n-c_ VDR-IRS			cancerous foci Δ_n-c_ VDR-IRS	
	Median (IQR)	p-Value	Median (IQR)		p-Value	Median (IQR)	p-Value		Median (IQR)	p-Value
*Molecular subtype of tumor * triple-negative breast cancer other	2 (-1-2) -1 (-4-0)	0.033 p^BH^ > 0.05	4 (2-4) 0 (0-2)		0.013 p^BH^ > 0.05	2 (2-3) 3 (1-6)	0.6		-1 (-2-2) 0.5 (-1-2)	0.2
*Vascular invasion * absent present	-1 (-2-2) -2 (-4-0)	0.023 p^BH^ > 0.05	0 (0-4) 1 (0-2)		0.9	2 (0-5) 3 (2-6)	0.3		1.5 (0-3.5) 0 (-2-2)	0.1
*Menopausal status* positive negative	-1 (-2-2) -3 (-5-0)	0.008 p^BH^ = 0.048	1 (0-4) 0 (0-2)		0.2	2 (1-5) 3 (2-6)	0.4		0.5 (-2-3) 0 (-1-2)	0.6

HER2, human epidermal growth factor receptor 2; IQR, interquartile ratio; RCB, residual cancer burden; VDR-IRS, vitamin D receptor-immunoreactive score.

Due to the fact that VDR-IRS is an ordinal variable, the U Mann-Whitney test was used to assess the differences between the groups. For each group, median and IQR was given. If p-Value for the coefficient was below 0.05, the Benjamini-Hochberg procedure was applied to verify if the relationship remains significant after correction for multiple comparisons. Complete analysis of differences between dichotomous groups is presented in **Supplementary Table S4**.

**Table 4:** Comparisons of groups according to VDR-IRS.

All cases (RCB class 0-III)										
	normal lobules cytoplasmic VDR-IRS		normal lobules nuclear VDR-IRS			immune cells infiltrate VDR-IRS			normal lobules Δ_n-c_ VDR-IRS	
	Median (IQR)	p-Value	Median (IQR)		p-Value	Median (IQR)	p-Value		Median (IQR)	p-Value
*RCB class * 0 I II III	4 (0-4) 3 (0-3) 3 (0-4) 3 (0-4)	0.1	6 (4-9) 6 (6-9) 3 (3-6) 6 (3-6)		0.036 p^BH^ > 0.05	4 (3-6) 6 (4-9) 4 (2-4) 4 (2-4)	0.08		3 (1.5-5) 6 (3-6) 2 (1-4) 2 (0.5-6)	0.1
*Grade after NAC * 0 1 2 3	4 (0-4)^*^ 3 (0-4) 2 (0-3)^*^ 4 (0-4)	0.022 p^BH^ > 0.05	6 (4-9) 6 (3-6) 5 (3-6) 3 (2-6)		0.1	4 (3-6) 4 (2-4) 4 (2-4) 4 (4-6)	0.1		3 (2-5) 2 (2-5) 3 (2-4) 2 (-2-2)	0.2
Cases without pathological complete response (RCB class > 0)										
	normal lobules cytoplasmic VDR-IRS		normal lobules nuclear VDR-IRS		immune cells infiltrate VDR-IRS		cancerous foci cytoplasmic VDR-IRS		cancerous foci nuclear VDR-IRS	
	Median (IQR)	p-Value	Median (IQR)	p-Value	Median (IQR)	p-Value	Median (IQR)	p-Value	Median (IQR)	p-Value
*RCB class * I II III	3 (0-3) 3 (0-4) 3 (0-4)	0.9	6 (6-9)^*^ 3 (3-6)^*^ 6 (3-6)	0.043 p^BH^ > 0.05	6 (4-9) 4 (2-4) 4 (2-4)	0.031 p^BH^ > 0.05	4 (4-8)^*^ 4 (0-4)^*^ 4 (2-4)	0.024 p^BH^ = 0.048	8 (2-9) 2 (1-4) 2 (1-6)	0.06
	Δ_nuclear_ VDR-IRS		Δ_cytoplasmic_ VDR-IRS			normal lobules Δ_n-c_ VDR-IRS			cancerous foci Δ_n-c_ VDR-IRS	
	Median (IQR)	p-Value	Median (IQR)		p-Value	Median (IQR)	p-Value		Median (IQR)	p-Value
*RCB class * I II III	2 (-2-3) -1 (-3-0) -1.5 (-4-1.5)	0.2	4 (4-5)^*,#^ 0 (0-3)^*^ 1 (0-2)^#^		0.005 p^BH^ = 0.01	6 (3-6)^*^ 2 (1-4)^*^ 2 (0.5-6)	0.042 p^BH^ > 0.05		2 (0-5) 0 (-1-2) 0 (-2-2)	0.2

Symbols (^*^,^#^) denote significant differences between groups in the post-hoc Dunn’s test. HER2, human epidermal growth factor receptor 2; IQR, interquartile ratio; RCB, residual cancer burden; VDR-IRS, vitamin D receptor-immunoreactive score.

Due to the non-normal distribution of the data the Kruskal-Wallis ANOVA was used to assess the differences between the groups. If any significant difference between groups was identified, the post-hoc Dunn’s test was used to find the pairs of group that differed. For each group, median and IQR was given. Complete analysis of differences between groups is presented in **Supplementary Table S5**.

Analysis of relationships between binomial variables showed that in the group with RCB class > 0, the cases with a low cytoplasmic VDR-IRS score for cancerous foci had a positive nodal status and invaded vessels much more frequently than the subjects with high cytoplasmic VDR-IRS in cancerous foci (65% vs. 0%, p^BH^ = 0.006 and 65% vs. 17%, p^BH^ = 0.032, respectively).

Ultimately, we propose a set of logistic regression models to evaluate the utility of VDR-IRS in the prediction of lymph node involvement and vascular invasion. Models were built for the entire study group and for subjects without a complete pathological response. After univariate analysis, optimal models were selected according to the Nagelkerke pseudo-R^2^, Hosmer-Lemeshow test, and ROC AUC for testing data. The set of parameters introduced for analysis included age of the subject, menopausal status, chemotherapy regimen, number of cycles, tumor-infiltrating lymphocyte density, Ki-67 expression, nuclear grade, HER2 status, ER and PR expression, and VDR-IRS.

In these logistic regression models, the increase in cytoplasmic VDR-IRS in normal lobules was associated with lower odds of vascular invasion for both the entire study group and a subpopulation of subjects with RCB > 0. However, a higher nuclear VDR-IRS of normal lobules was associated with increased odds of lymph node metastases in both the investigated models. According to the AUC for testing data after 10-fold cross-validation, models predicting vascular invasion had a better capability of making correct predictions than models for lymph node metastases.

All received models of logistic regression are presented in **Table 5**.

**Table 5:** Logistic regression models predicting the odds of vascular invasion (models 1 and 2) and the involvement of lymph nodes after NAC (models 3 and 4).

All cases (RCB 0-III)			Cases without pathological complete response (RCB > 0)		
Model 1 – vascular invasion^1^			Model 2 – vascular invasion^2^		
Parameter	OR (95%CI)	p-Value	Parameter	OR (95%CI)	p-Value
*Molecular subtype * triple-negative breast cancer other	17.71 (1.13-276.53) reference	0.04	*Molecular subtype* triple-negative breast cancer other	1.90 (0.19-19.06) reference	0.6
normal lobules cytoplasmic VDR-IRS (per 1 point)	0.63 (0.45-0.87)	0.005	normal lobules cytoplasmic VDR-IRS (per 1 point)	0.67 (0.46-0.99)	0.042
Ki-67 before NAC (per 1%)	1.05 (0.96-1.14)	0.3	cancerous foci cytoplasmic VDR-IRS (per 1 point)	0.95 (0.79-1.15)	0.6
Estrogen receptor expression (per 1%)	1.03 (1.01-1.05)	0.012	Estrogen receptor expression (per 1%)	1.01 (0.99-1.03)	0.2
Progesterone receptor expression (per 1%)	0.98 (0.96-1.01)	0.1	Grade (before NAC)	0.66 (0.22-1.90)	0.2
Grade (before NAC)	1.63 (0.13-21.16)	0.7	Chemotherapy cycles number	0.82 (0.69-0.97)	0.024
Chemotherapy cycles number	1.22 (0.74-2.02)	0.4	–	–	–

Model 3 – involvement of LNs after NAC^3^			Model 4 – involvement of LNs after NAC ^4^		
Parameter	OR (95%CI)	p-Value	Parameter	OR (95%CI)	p-Value
normal lobules nuclear VDR-IRS (per 1 point)^5^	4.49 (1.39-14.40)	0.012	*Histological type* other than NST NST	0.25 (0.05-1.19) reference	0.08
Ki-67 before NAC (per 1%)	1.15 (1.02-1.29)	0.018	normal lobules nuclear VDR-IRS^5^ (per 1 point)	2.24 (1.03-4.85)	0.041
Estrogen receptor expression (per 1%)	1.02 (1.00-1.04)	0.1	Tumor infiltrating lymphocytes (per 1%)	1.02 (0.67-1.56)	0.9
Progesterone receptor expression (per 1%)	0.99 (0.97-1.01)	0.3	Estrogen receptor expression (per 1%)	1.00 (0.96-1.05)	1.0
Chemotherapy cycles number^5^	5.19 (1.64-16.45)	0.005	Chemotherapy cycles number^5^	1.19 (0.76-1.88)	0.4

^1^ Nagelkerke pseudo-R^2^ = 0.53, Hosmer-Lemeshow test p-Value 9= 0.17, AUC for training data = 0.88, AUC for testing data with 10-fold cross validation = 0.81; ^2^ Nagelkerke pseudo-R^2^ = 0.31, Hosmer-Lemeshow test p-Value = 0.54, AUC for training data = 0.78, and AUC for testing data with 10-fold cross validation = 0.69; ^3^ Nagelkerke pseudo-R^2^ = 0.57, Hosmer-Lemeshow test p-Value = 0.81, AUC for training data = 0.88, and AUC for testing data with 10-fold cross validation = 0.83; ^4^ Nagelkerke pseudo-R^2^ = 0.42, Hosmer-Lemeshow test p-Value = 0.99, AUC for training data = 0.86, and AUC for testing data with 10-fold cross validation = 0.68; ^5^ significant interaction between nuclear VDR-IRS and NAC cycle number that was considered in models 3 and 4.

LN, lymph nodes; NAC, neoadjuvant chemotherapy; OR (95%CI), odds ratio with 95% confidence interval; VDR-IRS, vitamin D receptor-immunoreactive score.

## 4 Discussion

This observational study aimed to determine the relationships between clinicopathological characteristics of female patients with BC after NAC and the expression of VDR (measured as IRS) in tumor cells and their microenvironment. To avoid the recognition of dependencies that emerged only accidentally due to multiple testing, we performed the Benjamini-Hochberg procedure. We considered the results that retained a p-Value lower than 0.05 after this correction. However, the Benjamini-Hochberg procedure might also lead to false-negative results in the case of real but weak relationships (25), therefore, our results should be interpreted with reason.

In the analyses that covered all subjects in the study (i.e., RCB class 0-III), we found that the nuclear pool of VDR was reduced compared to the cytoplasmic pool in breast tissue of older participants.

Considering the subgroup with residual disease after NAC, more relationships were identified. The expression of VDR in the immune infiltrate increased in tumors with a higher proliferative potential (higher expression of Ki-67 before NAC) and was inversely correlated with the expression of ER. Notably, Ki-67 and ER showed a substantial negative correlation (R = -0.46, p< 0.0001). Women with a positive menopausal status had higher VDR-IRS in cancer cells than in the remaining breast tissue (compared to those without menopause). Interestingly, subjects with distinctly higher VDR-IRS in the tumor cell cytoplasm than in normal lobules had RCB-I more frequently than RCB-II or RCB-III. The aforementioned difference correlated with the number of NAC cycles.

High expression of VDR in the cytoplasm of tumor cells (IRS ≥ 8) appeared to be prevalent in cases without vascular invasion and lymph node metastases. However, our logistic regression models suggested that more valid predictions of these features could be made with VDR-IRS of the cytoplasm and nuclei of normal lobules, respectively.

The vitamin D receptor plays a multidirectional role in both physiology and disease. In the breast, it is normally synthesized in remarkable amounts in the epithelium, stromal tissues, and immune cells. Its most well-described functions are regulation of cell proliferation, differentiation, and apoptosis, as well as modulation of immune cell activity (26). VDR is directly involved in the metabolism of calcium and phosphorus, which is linked to both healthy breast function (i.e., lactation) and BC (tendency to metastasize to the bones) (27). Some researchers have claimed that VDR is responsible for autophagy in healthy mammary glands and might trigger autophagy in BC cells (28). Studies in mouse models have suggested that the lack of VDR in BC cells (but not in the surrounding stroma) is associated with a marked increase in metastatic potential (29).

The exact mechanisms that govern the synthesis of VDR involve regulation at the transcriptional and post-transcriptional levels (i.e., DNA methylation and microRNA) or at the protein level (phosphorylation of specific sites and various distributions across cellular compartments). Naturally, the majority of the biological effects of VDR are strictly dependent on the presence of its ligand, 1,25-(OH)_2_-D_3_. Intracellular hydroxylases (such as CYP24A1) that inactivate it are another important contributor to the effects of VDR (30).

The most biologically relevant actions of 1,25-(OH)_2_-D_3_ are mediated by the VDR. The receptor is a ligand-dependent transcription factor from the superfamily of nuclear receptors and can form an active heterodimer, mainly with one of the orphan retinoid X receptors (RXR) (31). Upon binding of the ligand, the VDR-RXR heterodimer (other combinations, such as homodimers, VDR-thyroid hormone receptor (VDR-TR), or VDR-retinoic acid receptor (VDR-RAR) are less active) targets specific sequences of DNA (i.e., VDR response element) and modulates the expression of a given gene, usually by recruitment of coactivators (32). In contrast, the lack of a ligand or the presence of an antagonist would facilitate the accumulation of corepressors. Notably, VDR does not dimerize with ER and PR; however, evidence suggests that its interactions with steroid hormone metabolism are caused by its ability to downregulate aromatase synthesis (33).

Although VDR is primarily a nuclear receptor, its cytoplasmic pool can exert independent actions, that is, through interaction with the MEK/ERK pathway, after activation of c-Raf (34).

During mutagenesis, which leads to the development of BC, both the expression and structure of VDR are affected. Extensive methylation of the VDR gene sequence has been reported to decrease its synthesis to 20% of the level observed in neighboring normal cells. Atypical truncated structural variants of VDR could form in BC cells (35).

A superficial overview of these data suggests a simple beneficial role for 1,25-(OH)_2_-D_3_ and VDR in the context of BC. There is robust evidence that supports this statement (36), however, some analyzes found no such association or even opposite results (37, 38). Further complexity of the topic is imposed by the presence of several polymorphisms in the VDR gene, some of which are associated with risk of development and progression of BC, such as CDX2, FOK1, BSM1, APA1, BGL1, TAQ1 and POLY(A) (39). Finally, in the context of NAC, it is reasonable to expect that in addition to the usual effect of VDR, there would be some interaction with a wide range of chemotherapy consequences. For example, cellular models have shown that VDR might potentiate the action of tamoxifen by downregulating the Wnt/β-catenin pathway in MCF-7 cancer stem cells (40).

We did not identify studies on VDR tissue expression in patients treated with NAC prior to BC surgery. Therefore, further discussion might be related only to studies that investigated serum vitamin D_3_ levels in women with BC who underwent NAC or studies that assessed VDR expression in BC tissues without preceding NAC. Hence, we could not thoroughly determine the external validity of our results.

A study by Viala et al. showed that women with vitamin D_3_ deficiency had lower odds of pCR (16). Recently, Tokunaga et al. reported that lower serum vitamin D_3_ levels were associated with a shorter time to distant recurrence, although not with pCR (17). Previous studies have not found any of these relationships in patients after NAC (41, 42).

Studies that investigated the role of VDR in BC tissues were summarized in a meta-analysis conducted by Xu et al. in 2020 (15). The total expression of VDR in the nuclei and cytoplasm was associated with improved overall survival, and heterogeneity across the studies was not caused by factors such as histological and molecular type, staining location, or cutoff value (for dichotomous analysis). However, most of these studies assessed only the nuclear expression of VDR (43–47). Two studies incorporated the evaluation of cytoplasmic VDR; however, one used data only from TNBC and determined VDR dichotomously as positive or negative (48), while the second considered only postmenopausal women (23).

The inverse relationship between age and Δ_n-c_ VDR-IRS in normal lobules might reflect the shift toward the cytoplasmic compartment caused by lowered 25-(OH)-D_3_ concentrations in the serum of older participants. Interestingly, this relationship was not observed in tumor cells. The expression of cytoplasmic VDR was more prominent than the nuclear amount of the receptor, and this may be due to the reduced capacity of BC cells to uptake 25-(OH)-D_3_ and also due to the altered synthesis of active 1,25-(OH)_2_-D_3_ due to down-regulation of CYP27B1 hydroxylase (14).

The higher the number of NAC cycles, the larger the VDR-IRS of tumor cell cytoplasm compared to normal cells, and a similar, though weaker, effect was observed for nuclear VDR-IRS. The VDR-IRS of cancer cells increased with the number of NAC cycles, and the expression of receptors in the cytoplasm of normal cells decreased. One possible explanation is that chemotherapy leads to an increase in the more active nuclear pool of VDR in cancer cells (which, as mentioned previously, disturbs the balance between cytoplasmic and nuclear VDR) and reduces cytoplasmic VDR in normal cells. Importantly, the number of cycles of NAC did not correlate with RCB (considering classes I-III), therefore, a higher Δ_cytoplasmic_ VDR-IRS in subjects with RCB-I (compared to RCB-II or III) is likely independent of it. This is consistent with data presented in previous research, where a higher cytoplasmic VDR-IRS was observed in tumors with a better prognosis (44), although a similar result was observed for nuclear VDR-IRS. When we considered VDR-IRS as a dichotomous variable, only high cytoplasmic VDR-IRS in cancer cells was associated with a low rate of nodal metastases and vascular invasion, consistent with the observations above.

Finally, our logistic regression models revealed an interesting relationship between VDR-IRS from the nuclei and cytoplasm of normal mammary cells and the odds of vascular invasion and lymph node involvement. These relationships were recognized only in the multivariate analysis, which unveils the complexity of the relationships between numerous factors responsible for NAC effectiveness in BC. Normal mammary cells are part of the tumor microenvironment and therefore are affected in the process of metastasis (49), and this is why changes in their molecular signature might be a reasonable source of information on cancer potential to metastasize. Generally, a higher cytoplasmic VDR-IRS is associated with decreased odds of vascular invasion. An interesting possibility is the influence of cytoplasmic VDR on the Notch pathway in normal mammary cells, and thus the remodeling of the extracellular matrix (49, 50). On the contrary, higher nuclear VDR-IRS increased the odds of lymph node involvement, which could suggest direct involvement of VDR in the way the tumor microenvironment prevents (or potentializes) cancer cell invasion. Notably, there was a significant interaction between the nuclear VDR-IRS of normal lobules and NAC cycles; thus, the observed relationship might be unique to tumors treated with chemotherapy.

This study had some limitations. First, due to the retrospective design of the study, the cause-and-effect relationships between variables cannot be rigorously claimed. Second, the expression of VDR is affected by the current amount of available 25-(OH)-D_3_, and we were not able to adjust for this variable.

## 5 Conclusions

In this study, we showed that the expression of VDR in tumor cells and its microenvironment is related to various clinicopathological characteristics of BC (including those of well-known prognostic meaning) after NAC. Further exploration of these findings is warranted, with the potential to consider VDR as a new prognostic marker in BC or even as a candidate target for new therapies.

## Data Availability

The raw data supporting the conclusions of this article will be made available by the authors, without undue reservation.
