# Supplementary material for: Vitamin D receptor is associated with prognostic characteristics of breast cancer after neoadjuvant chemotherapy—an observational study
Source: Front Oncol. 2024 Oct 1;14:1458124. doi: 10.3389/fonc.2024.1458124 (PMC11476186; doi:10.3389/fonc.2024.1458124)
Supplement: Supplementary file 1 [file DataSheet1.docx]

**Supplementary Table S1.** Antibodies used for immunohistochemical staining.

| **Primary antibody** | **Source clone** | **Dilution** | **Antigen retrieval** | **Time**  **of incubation** | **Producer** | **Detection system** |
| --- | --- | --- | --- | --- | --- | --- |
| VDR | D2K6W | 2:100 | EDTA | 40 min | Cell Signaling Technology | ultraView Universal DAB  Detection Kit (Roche Ventana) |
| ER | SP1 | RTU | EDTA | 16 min | Roche | ultraView Universal DAB  Detection Kit (Roche Ventana) |
| PR | 1E2 | RTU | EDTA | 16 min | Roche | ultraView Universal DAB  Detection Kit (Roche Ventana) |
| Ki-67 | MIB-1 | RTU | EDTA | 20 min | Dako, USA | ultraView Universal DAB  Detection Kit (Roche Ventana) |
| HER2/neu | 4B5 | RTU | Citrate | 16 min | Roche | ultraView Universal DAB  Detection Kit (Roche Ventana) |

**Supplementary Table S2.** Detailed list of the drugs used for neoadjuvant chemotherapy (or chemoimmunotherapy). Data are presented as absolute and relative frequencies: N (%).

| **Drug** | **All cases**  **N (%)** | **Cases without pathological complete response**  **(RCB class > 0)**  **N (%)** |
| --- | --- | --- |
| Paclitaxel | 44 (40%) | 26 (36%) |
| Docetaxel | 63 (57%) | 43 (59%) |
| Platinum(IV) derivatives | 30 (30%) | 12 (16%) |
| Carboplatin | 29 (26%) | 11 (15%) |
| Doxorubicin | 98 (88%) | 63 (86%) |
| Epirubicin | 4 (4%) | 3 (4%) |
| Cyclophosphamide | 98 (88%) | 62 (85%) |
| Trastuzumab | 14 (13%) | 4 (5%) |
| Trastuzumab + pertuzumab | 21 (19%) | 17 (23%) |

**Supplementary Table S3.** Correlations between VDR-IRS and the clinicopathological characteristics of BC. The table presents results separately for all investigated subjects (VDR-IRS of normal lobules and immune cells infiltrate) and for subjects without complete pathological response (VDR-IRS also for cancerous foci). Furthermore, correlations are presented with secondary variables received by subtracting the original VDR-IRS values. Spearman correlation coefficient R was used to represent the direction and magnitude of the relationship. If the p-Value for the coefficient was below 0.05, the Benja-mini-Hochberg procedure was applied to verify whether the correlation remains significant after correction for multiple comparisons.

| **All cases (RCB class 0-3)** | | | | | | | | | | | | | | | | | | | | | | | | | | | | | | | | | | | | | | |
| --- | --- | --- | --- | --- | --- | --- | --- | --- | --- | --- | --- | --- | --- | --- | --- | --- | --- | --- | --- | --- | --- | --- | --- | --- | --- | --- | --- | --- | --- | --- | --- | --- | --- | --- | --- | --- | --- | --- |
|  | | | | | normal lobules  cytoplasmic VDR-IRS | | | | | | | | | normal lobules  nuclear VDR-IRS | | | | | | | | | | immune cells infiltrate VDR-IRS | | | | | | | | normal lobules  Δ_n-c_ VDR-IRS | | | | | | |
|  | |  | | | Spearman R | | | | p-Value | | | | | Spearman R | | | | p-Value | | | | | | Spearman R | | | | p-Value | | | |  | | | |  | | |
| age (years) | | | | | 0.20 | | | | 0.047  p^BH^ > 0.05 | | | | | -0.16 | | | | 0.1 | | | | | | -0.03 | | | | 0.7 | | | | -0.31 | | | | 0.0015  p^BH^ = 0.014 | | |
| TIL (%) | | | | | -0.02 | | | | 0.9 | | | | | -0.19 | | | | 0.051 | | | | | | 0.09 | | | | 0.4 | | | | -0.17 | | | | 0.1 | | |
| Ki-67 before NAC (%) | | | | | -0.05 | | | | 0.6 | | | | | -0.04 | | | | 1.0 | | | | | | 0.20 | | | | 0.038  p^BH^ > 0.05 | | | | 0.05 | | | | 0.6 | | |
| RCB | | | | | -0.20 | | | | 0.039  p^BH^ > 0.05 | | | | | -0.15 | | | | 0.1 | | | | | | -0.13 | | | | 0.1 | | | | -0.04 | | | | 0.7 | | |
| estrogen receptor (%) | | | | | -0.02 | | | | 0.9 | | | | | 0.01 | | | | 0.9 | | | | | | -0.15 | | | | 0.1 | | | | 0.04 | | | | 0.7 | | |
| progesterone receptor (%) | | | | | -0.05 | | | | 0.6 | | | | | -0.09 | | | | 0.4 | | | | | | -0.06 | | | | 0.5 | | | | -0.03 | | | | 0.8 | | |
| grade after NAC | | | | | -0.16 | | | | 0.1 | | | | | -0.25 | | | | 0.016  p^BH^ > 0.05 | | | | | | -0.03 | | | | 0.8 | | | | -0.14 | | | | 0.2 | | |
| tumor size (cm) | | | | | -0.17 | | | | 0.09 | | | | | -0.13 | | | | 0.2 | | | | | | -0.12 | | | | 0.2 | | | | -0.04 | | | | 0.7 | | |
| chemotherapy cycles number | | | | | -0.21 | | | | 0.032  p^BH^ > 0.05 | | | | | -0.03 | | | | 0.8 | | | | | | -0.07 | | | | 0.5 | | | | 0.16 | | | | 0.1 | | |
| **Cases without pathological complete response (RCB class > 0)** | | | | | | | | | | | | | | | | | | | | | | | | | | | | | | | | | | | | | | |
|  |  | | | normal lobules  cytoplasmic VDR-IRS | | | | | | | | normal lobules  nuclear VDR-IRS | | | | | | | | immune cells infiltrate  VDR-IRS | | | | | | cancerous foci  cytoplasmic VDR-IRS | | | | | | | | cancerous foci  nuclear VDR-IRS | | | | |
|  |  | | Spearman R | | | p-Value | | | | | Spearman R | | | | | p-Value | | | | Spearman R | | | p-Value | | | Spearman R | | | | p-Value | | | | Spearman R | | | | p-Value |
| age (years) | | | | | 0.18 | | 0.2 | | | -0.20 | | | | | 0.1 | | | | >0.01 | | 1.0 | | | | 0.03 | | | | 0.8 | | | | 0.14 | | | | 0.5 | |
| TIL (%) | | | | | -0.04 | | 0.8 | | | -0.23 | | | | | 0.06 | | | | 0.03 | | 0.8 | | | | -0.10 | | | | 0.4 | | | | 0.07 | | | | 0.6 | |
| Ki-67 before NAC (%) | | | | | -0.05 | | 0.7 | | | -0.04 | | | | | 0.7 | | | | 0.34 | | 0.004  p^BH^ = 0.036 | | | | 0.05 | | | | 0.7 | | | | 0.13 | | | | 0.3 | |
| RCB | | | | | -0.03 | | 0.8 | | | -0.06 | | | | | 0.7 | | | | -0.20 | | 0.1 | | | | -0.12 | | | | 0.3 | | | | -0.16 | | | | 0.2 | |
| estrogen receptor (%) | | | | | 0.13 | | 0.3 | | | 0.20 | | | | | 0.9 | | | | -0.30 | | 0.011  p^BH^ = 0.0495 | | | | -0.13 | | | | 0.3 | | | | -0.22 | | | | 0.07 | |
| progesterone receptor (%) | | | | | 0.08 | | 0.5 | | | -0.14 | | | | | 0.3 | | | | -0.20 | | 0.1 | | | | -0.22 | | | | 0.07 | | | | -0.21 | | | | 0.08 | |
| grade after NAC | | | | | 0.18 | | 0.2 | | | -0.16 | | | | | 0.2 | | | | 0.22 | | 0.09 | | | | -0.07 | | | | 0.6 | | | | 0.12 | | | | 0.3 | |
| tumor size (cm) | | | | | 0.06 | | 0.6 | | | 0.02 | | | | | 0.9 | | | | -0.14 | | 0.3 | | | | -0.16 | | | | 0.2 | | | | -0.21 | | | | 0.08 | |
| chemotherapy cycles number | | | | | -0.33 | | 0.008  p^BH^ > 0.05 | | | -0.05 | | | | | 0.7 | | | | -0.06 | | 0.6 | | | | 0.18 | | | | 0.1 | | | | 0.25 | | | | 0.037  p^BH^ > 0.05 | |
|  | | | | |  | |  | | |  | | | | |  | | | |  | |  | | | |  | | | |  | | | |  | | | |  | |
|  | | | | | Δ_nuclear_ VDR-IRS | | | | | | | | Δ_cytoplasmic_ VDR-IRS | | | | | | | | | normal lobules  Δ_n-c_ VDR-IRS | | | | | | | | | cancerous foci  Δ_n-c_ VDR-IRS | | | | | | | |
|  | | | | | Spearman R | | | p-Value | | | | | Spearman R | | | | p-Value | | | | | Spearman R | | | | | p-Value | | | | Spearman R | | | | p-Value | | | |
| age (years) | | | | | 0.33 | | | 0.008  p^BH^ > 0.05 | | | | | -0.07 | | | | 0.6 | | | | | -0.32 | | | | | 0.009  p^BH^ > 0.05 | | | | 0.05 | | | | 0.7 | | | |
| TIL (%) | | | | | 0.17 | | | 0.2 | | | | | 0.01 | | | | 0.9 | | | | | -0.17 | | | | | 0.2 | | | | 0.03 | | | | 0.8 | | | |
| Ki-67 before NAC (%) | | | | | 0.14 | | | 0.3 | | | | | 0.09 | | | | 0.5 | | | | | -0.08 | | | | | 0.5 | | | | -0.17 | | | | 0.1 | | | |
| RCB | | | | | -0.17 | | | 0.2 | | | | | -0.10 | | | | 0.4 | | | | | -0.01 | | | | | 0.9 | | | | 0.08 | | | | 0.5 | | | |
| estrogen receptor (%) | | | | | -0.31 | | | 0.013  p^BH^ > 0.05 | | | | | -0.21 | | | | 0.1 | | | | | -0.01 | | | | | 1.0 | | | | -0.16 | | | | 0.2 | | | |
| progesterone receptor (%) | | | | | -0.19 | | | 0.4 | | | | | -0.21 | | | | 0.1 | | | | | -0.10 | | | | | 0.4 | | | | -0.07 | | | | 0.5 | | | |
| grade after NAC | | | | | 0.12 | | | 0.4 | | | | | -0.18 | | | | 0.2 | | | | | -0.25 | | | | | 0.1 | | | | 0.10 | | | | 0.4 | | | |
| tumor size (cm) | | | | | -0.31 | | | 0.011  p^BH^ > 0.05 | | | | | -0.23 | | | | 0.1 | | | | | -0.09 | | | | | 0.5 | | | | -0.17 | | | | 0.1 | | | |
| chemotherapy cycles number | | | | | 0.29 | | | 0.017  p^BH^ > 0.05 | | | | | 0.41 | | | | 0.0006  p^BH^ = 0.005 | | | | | 0.20 | | | | | 0.1 | | | | 0.17 | | | | 0.1 | | | |

**Abbreviations:** BC—breast cancer; HER2—human epidermal growth factor receptor 2; NAC—neoadjuvant chemotherapy; RCB—residual cancer burden; TIL—tumor infiltrating lymphocytes; VDR-IRS—vitamin D receptor-immunoreactive score.

**Supplementary Table S4.** Comparisons of dichotomous groups according to VDR-IRS. Due to the fact that VDR-IRS is an ordinal variable, the U Mann-Whitney test was used to assess the differences between the groups. For each group, median and IQR was given. If p-Value for the coefficient was below 0.05, the Benjamini-Hochberg procedure was applied to verify if the relationship remains significant after correction for multiple comparisons.

| **All cases (RCB class 0-3)** | | | | | | | | | | | | | | | | | | | | | | | | | | | | | | | | | |
| --- | --- | --- | --- | --- | --- | --- | --- | --- | --- | --- | --- | --- | --- | --- | --- | --- | --- | --- | --- | --- | --- | --- | --- | --- | --- | --- | --- | --- | --- | --- | --- | --- | --- |
|  | | | | | normal lobules  cytoplasmic VDR-IRS | | | | | | | normal lobules  nuclear VDR-IRS | | | | | | | | immune cells infiltrate VDR-IRS | | | | | | | normal lobules  Δ_n-c_ VDR-IRS | | | | | | |
|  | |  | | | Median (IQR) | | | p-Value | | | | Median (IQR) | | | p-Value | | | | | Median (IQR) | | | p-Value | | | | Median (IQR) | | | | p-Value | | |
| *Histological type of tumor*  no-special type  other | | | | | 3 (0-4)  3 (0-4) | | | 0.9 | | | | 6 (3-6)  6 (3-9) | | | 0.2 | | | | | 4 (2-6)  4 (4-4) | | | 1.0 | | | | 3 (1-5)  3 (2-6) | | | | 0.4 | | |
| *RCB class*  0  1-3 | | | | | 4 (0-4)  3 (0-4) | | | 0.023  p^BH^ > 0.05 | | | | 6 (4-9)  6 (3-6) | | | 0.1 | | | | | 4 (3-6)  4 (2-6) | | | 0.5 | | | | 3 (2-5)  3 (1-5) | | | | 0.7 | | |
| *Molecular subtype of tumor*  triple-negative breast cancer other | | | | | 3 (0-4)  2.5 (0-4) | | | 0.5 | | | | 6 (3-8)  6 (2-6) | | | 0.3 | | | | | 4 (3-6)  4 (2-4) | | | 0.4 | | | | 3 (1-5)  2 (2-5) | | | | 0.7 | | |
| *HER2 status*  positive  negative | | | | | 4 (0-4)  3 (0-4) | | | 0.3 | | | | 6 (4-6)  6 (3-8) | | | 0.3 | | | | | 4 (3-6)  4 (2-4) | | | 0.1 | | | | 2.5 (1-5)  3 (1-5) | | | | 1.0 | | |
| *ypT*  0 or is  1-4 | | | | | 4 (0-4)  3 (0-4) | | | 0.01  p^BH^ > 0.05 | | | | 6 (4-9)  6 (3-6) | | | 0.1 | | | | | 4 (2.5-6)  4 (2-4) | | | 0.5 | | | | 3 (2-5)  3 (1-5) | | | | 1.0 | | |
| *ypN*  0  1-3 | | | | | 4 (0-4)  3 (0-4) | | | 0.2 | | | | 6 (3-9)  6 (3-6) | | | 0.2 | | | | | 4 (3-6)  4 (2-6) | | | 0.5 | | | | 3 (2-5)  2 (1-5) | | | | 0.5 | | |
| *Vascular invasion*  absent  present | | | | | 4 (0-4)  2 (0-4) | | | 0.01  p^BH^ > 0.05 | | | | 6 (3-9)  6 (3-6) | | | 0.4 | | | | | 4 (3-6)  4 (2-6) | | | 0.5 | | | | 2 (1-5)  3 (2-6) | | | | 0.5 | | |
| *Menopausal status*  positive  negative | | | | | 3 (0-4)  2 (0-4) | | | 0.6 | | | | 6 (3-6)  6 (3-8) | | | 0.3 | | | | | 4 (3-6)  4 (2-6) | | | 0.7 | | | | 2 (1-5)  3 (2-6) | | | | 0.1 | | |
| **Cases without pathological complete response (RCB class > 0)** | | | | | | | | | | | | | | | | | | | | | | | | | | | | | | | | | |
|  |  | | | normal lobules  cytoplasmic VDR-IRS | | | | | | | normal lobules  nuclear VDR-IRS | | | | | | immune cells infiltrate  VDR-IRS | | | | | cancerous foci  cytoplasmic VDR-IRS | | | | | | | cancerous foci  nuclear VDR-IRS | | | | |
|  |  | | Median (IQR) | | | p-Value | | | | Median (IQR) | | | | p-Value | | | Median (IQR) | | p-Value | | | Median (IQR) | | | p-Value | | | | Median (IQR) | | | | p-Value |
| *Histological type of tumor*  no-special type  other | | | | | 3 (0-4)  3 (0-4) | | 0.9 | | 6 (3-6)  6 (3-8) | | | | 0.5 | | | 4 (2-6)  4 (3-4) | | 0.7 | | | 4 (1-4)  4 (1-4) | | | 0.9 | | | | 2 (1-6)  3 (1-6) | | | | 0.8 | |
| *Molecular subtype of tumor*  triple-negative breast cancer other | | | | | 0 (0-3)  3 (0-4) | | 0.2 | | 3 (2-6)  6 (3-6) | | | | 0.1 | | | 4 (4-4)  4 (2-6) | | 0.8 | | | 4 (4-4)  4 (0-4) | | | 0.03 | | | | 2.5 (2-8)  2.5 (1-6) | | | | 0.6 | |
| *HER2 status*  positive  negative | | | | | 3 (0-4)  3 (0-4) | | 0.4 | | 6 (3-6)  6 (3-6) | | | | 0.5 | | | 4 (4-6)  4 (2-4) | | 0.1 | | | 3.5 (1-4)  4 (1-4) | | | 0.3 | | | | 2.5 (1.5-7)  2 (1-6) | | | | 0.7 | |
| *ypN*  0  1-3 | | | | | 3 (0-4)  3 (0-4) | | 1.0 | | 6 (3-8)  6 (3-6) | | | | 0.6 | | | 4 (4-4)  4 (2-6) | | 0.6 | | | 4 (0-4)  4 (2-4) | | | 1.0 | | | | 3 (1-6)  2 (1-6) | | | | 1.0 | |
| *Vascular invasion*  absent  present | | | | | 3 (0-4)  2 (0-4) | | 0.1 | | 6 (3-6)  6 (3-6) | | | | 0.9 | | | 4 (3-4)  4 (2-6) | | 0.8 | | | 4 (2-4)  4 (0-4) | | | 0.3 | | | | 4 (2-8)  2 (1-4) | | | | 0.031  p^BH^ > 0.05 | |
| *Menopausal status*  positive  negative | | | | | 3 (0-4)  2 (0-4) | | 0.8 | | 6 (3-6)  6 (3-8) | | | | 0.5 | | | 4 (3.5-4)  4 (2-6) | | 0.4 | | | 4 (2.5-4)  3.5 (0-4) | | | 0.1 | | | | 3.5 (2-7)  2 (1-4) | | | | 0.0457  p^BH^ > 0.05 | |
|  | | | | | Δ_nuclear_ VDR-IRS | | | | | | | Δ_cytoplasmic_ VDR-IRS | | | | | | | | normal lobules  Δ_n-c_ VDR-IRS | | | | | | cancerous foci  Δ_n-c_ VDR-IRS | | | | | | | |
|  | | | | | Median (IQR) | | | p-Value | | | | Median (IQR) | | | p-Value | | | | | Median (IQR) | | | p-Value | | | Median (IQR) | | | | p-Value | | | |
| *Histological type of tumor*  no-special type  other | | | | | -1 (-3-2)  -3 (-5-0) | | | 0.2 | | | | 1(0-4)  0 (0-4) | | | 1.0 | | | | | 3 (1-5)  3 (2-6) | | | 0.6 | | | 1 (-1-3)  0 (-2-2) | | | | 0.4 | | | |
| *Molecular subtype of tumor*  triple-negative breast cancer other | | | | | 2 (-1-2)  -1 (-4-0) | | | 0.033  p^BH^ > 0.05 | | | | 4 (2-4)  0 (0-2) | | | 0.013  p^BH^ > 0.05 | | | | | 2 (2-3)  3 (1-6) | | | 0.6 | | | -1 (-2-2)  0.5 (-1-2) | | | | 0.2 | | | |
| *HER2 status*  positive  negative | | | | | -1.5 (-4-1)  -1 (-3-1) | | | 0.9 | | | | 0 (0-1)  1 (0-4) | | | 0.2 | | | | | 2 (1.5-5.5)  3 (1-5) | | | 0.9 | | | 2 (-1-3)  0 (-2-2) | | | | 0.2 | | | |
| *ypN*  0  1-3 | | | | | -1 (-3-1)  -1 (-4-1.5) | | | 0.9 | | | | 0.5 (0-4)  0.5 (0-2) | | | 0.7 | | | | | 3 (2-6)  2 (1-5) | | | 0.4 | | | 0 (0-3)  0 (-2-2) | | | | 0.5 | | | |
| *Vascular invasion*  absent  present | | | | | -1 (-2-2)  -2 (-4-0) | | | 0.023  p^BH^ > 0.05 | | | | 0 (0-4)  1 (0-2) | | | 0.9 | | | | | 2 (0-5)  3 (2-6) | | | 0.3 | | | 1.5 (0-3.5)  0 (-2-2) | | | | 0.1 | | | |
| *Menopausal status*  positive  negative | | | | | -1 (-2-2)  -3 (-5-0) | | | 0.008  p^BH^ = 0.48 | | | | 1 (0-4)  0 (0-2) | | | 0.2 | | | | | 2 (1-5)  3 (2-6) | | | 0.4 | | | 0.5 (-2-3)  0 (-1-2) | | | | 0.6 | | | |

**Abbreviations:** HER2—human epidermal growth factor receptor 2; IQR—interquartile ratio; RCB—residual cancer burden; VDR-IRS—vitamin D receptor-immunoreactive score.

**Supplementary Table S5.** Comparisons of groups according to VDR-IRS. Due to the non-normal distribution of the data the Kruskal-Wallis ANOVA was used to assess the differences between the groups. If any significant difference between groups was identified, the post-hoc Dunn’s test was used to find the pairs of group that differed. For each group, median and IQR was given.

| **All cases (RCB class 0-3)** | | | | | | | | | | | | | | | | | | | | | | | | | | | | | | | | | |
| --- | --- | --- | --- | --- | --- | --- | --- | --- | --- | --- | --- | --- | --- | --- | --- | --- | --- | --- | --- | --- | --- | --- | --- | --- | --- | --- | --- | --- | --- | --- | --- | --- | --- |
|  | | | | | normal lobules  cytoplasmic VDR-IRS | | | | | | | normal lobules  nuclear VDR-IRS | | | | | | | | immune cells infiltrate VDR-IRS | | | | | | | normal lobules  Δ_n-c_ VDR-IRS | | | | | | |
|  | |  | | | Median (IQR) | | | p-Value | | | | Median (IQR) | | | p-Value | | | | | Median (IQR) | | | p-Value | | | | Median (IQR) | | | | p-Value | | |
| *Molecular subtype of tumor*  luminal A  luminal B HER2-  luminal B HER2+  non-luminal HER2+  triple-negative breast cancer | | | | | 0 (0-4)  3 (0-4)  3.5 (0-4)  4 (0-4)  2.5 (0-4) | | | 0.8 | | | | 6 (6-9)  5 (3-8)  5 (3-6)  6 (6-9)  6 (2-6) | | | 0.3 | | | | | 3 (2-5)  4 (2-4)  6 (4-6)  4 (3-6)  4 (2-4) | | | 0.4 | | | | 6 (2-9)  3 (0.5-5)  2 (0-5)  3 (2-5)  2 (2-5) | | | | 0.5 | | |
| *RCB class*  0  1  2  3 | | | | | 4 (0-4)  3 (0-3)  3 (0-4)  3 (0-4) | | | 0.1 | | | | 6 (4-9)  6 (6-9)  3 (3-6)  6 (3-6) | | | 0.036  p^BH^ > 0.05 | | | | | 4 (3-6)  6 (4-9)  4 (2-4)  4 (2-4) | | | 0.08 | | | | 3 (1.5-5)  6 (3-6)  2 (1-4)  2 (0.5-6) | | | | 0.1 | | |
| *Grade after NAC*  0  1  2  3 | | | | | 4 (0-4)^*^  3 (0-4)  2 (0-3)^*^  4 (0-4) | | | 0.022  p^BH^ > 0.05 | | | | 6 (4-9)  6 (3-6)  5 (3-6)  3 (2-6) | | | 0.1 | | | | | 4 (3-6)  4 (2-4)  4 (2-4)  4 (4-6) | | | 0.1 | | | | 3 (2-5)  2 (2-5)  3 (2-4)  2 (-2-2) | | | | 0.2 | | |
| **Cases without pathological complete response (RCB class > 0)** | | | | | | | | | | | | | | | | | | | | | | | | | | | | | | | | | |
|  |  | | | normal lobules  cytoplasmic VDR-IRS | | | | | | | normal lobules  nuclear VDR-IRS | | | | | | immune cells infiltrate  VDR-IRS | | | | | cancerous foci  cytoplasmic VDR-IRS | | | | | | | cancerous foci  nuclear VDR-IRS | | | | |
|  |  | | Median (IQR) | | | p-Value | | | | Median (IQR) | | | | p-Value | | | Median (IQR) | | p-Value | | | Median (IQR) | | | p-Value | | | | Median (IQR) | | | | p-Value |
| *Molecular subtype of tumor*  luminal A  luminal B HER2-  luminal B HER2+  non-luminal HER2+  triple-negative breast cancer | | | | | 0 (0-4)  3 (0-4)  3 (1.5-4)  3 (0-4)  0 (0-3) | | 0.6 | | 6 (6-9)  4 (3-8)  6 (3-7)  6 (4-6)  3 (2-6) | | | | 0.3 | | | 3 (2-5)  4 (2-4)  6 (4-6)  4 (2-4)  4 (4-4) | | 0.041  p^BH^ > 0.05 | | | 2 (0-4)  4 (0-4)  3 (1-4)  3 (1-4)  4 (4-4) | | | 0.2 | | | | 3 (1-6)  2 (1-4)  4 (2-8)  2 (1-6)  2.5 (2-8) | | | | 0.7 | |
| *RCB class*  1  2  3 | | | | | 3 (0-3)  3 (0-4)  3 (0-4) | | 0.9 | | 6 (6-9)^*^  3 (3-6)^*^  6 (3-6) | | | | 0.043  p^BH^ > 0.05 | | | 6 (4-9)  4 (2-4)  4 (2-4) | | 0.031  p^BH^ > 0.05 | | | 4 (4-8)^*^  4 (0-4)^*^  4 (2-4) | | | 0.024  p^BH^ = 0.048 | | | | 8 (2-9)  2 (1-4)  2 (1-6) | | | | 0.06 | |
|  | | | | |  | |  | |  | | | |  | | |  | |  | | |  | | |  | | | |  | | | |  | |
|  | | | | | Δ_nuclear_ VDR-IRS | | | | | | | Δ_cytoplasmic_ VDR-IRS | | | | | | | | normal lobules  Δ_n-c_ VDR-IRS | | | | | | | cancerous foci  Δ_n-c_ VDR-IRS | | | | | | |
|  | | | | | Median (IQR) | | | p-Value | | | | Median (IQR) | | | p-Value | | | | | Median (IQR) | | | p-Value | | | | Median (IQR) | | | | p-Value | | |
| *Molecular subtype of tumor*  luminal A  luminal B HER2-  luminal B HER2+  non-luminal HER2+  triple-negative breast cancer | | | | | -4 (-9-2)  -1 (-3-0)  -1 (-2.5-3)  -3 (-4-0)  2 (-1-2) | | | 0.2 | | | | 0 (0-4)  0 (0-4)  0 (-1.5-2.5)  0 (0-1)  4 (2-4) | | | 0.1 | | | | | 6 (2-9)  3 (0-5)  2 (0-6)  3 (2-5)  2 (2-3) | | | 0.6 | | | 2 (-1-4)  0 (-2-2)  2 (0-3)  1 (-1-3)  -1 (-2-2) | | | | 0.3 | | | |
| *RCB class*  1  2  3 | | | | | 2 (-2-3)  -1 (-3-0)  -1.5 (-4-1.5) | | | 0.2 | | | | 4 (4-5)^*,#^  0 (0-3)^*^  1 (0-2)^#^ | | | 0.005  p^BH^ = 0.01 | | | | | 6 (3-6)^*^  2 (1-4)^*^  2 (0.5-6) | | | 0.042  p^BH^ > 0.05 | | | 2 (0-5)  0 (-1-2)  0 (-2-2) | | | | 0.2 | | | |

Symbols (^*^,^#^) denote significant differences between groups in post-hoc Dunn’s test. **Abbreviations:** HER2—human epidermal growth factor receptor 2; IQR—interquartile ratio; RCB—residual cancer burden; VDR-IRS—vitamin D receptor-immunoreactive score.

**Supplementary Figure S1.** Study flow-chart.


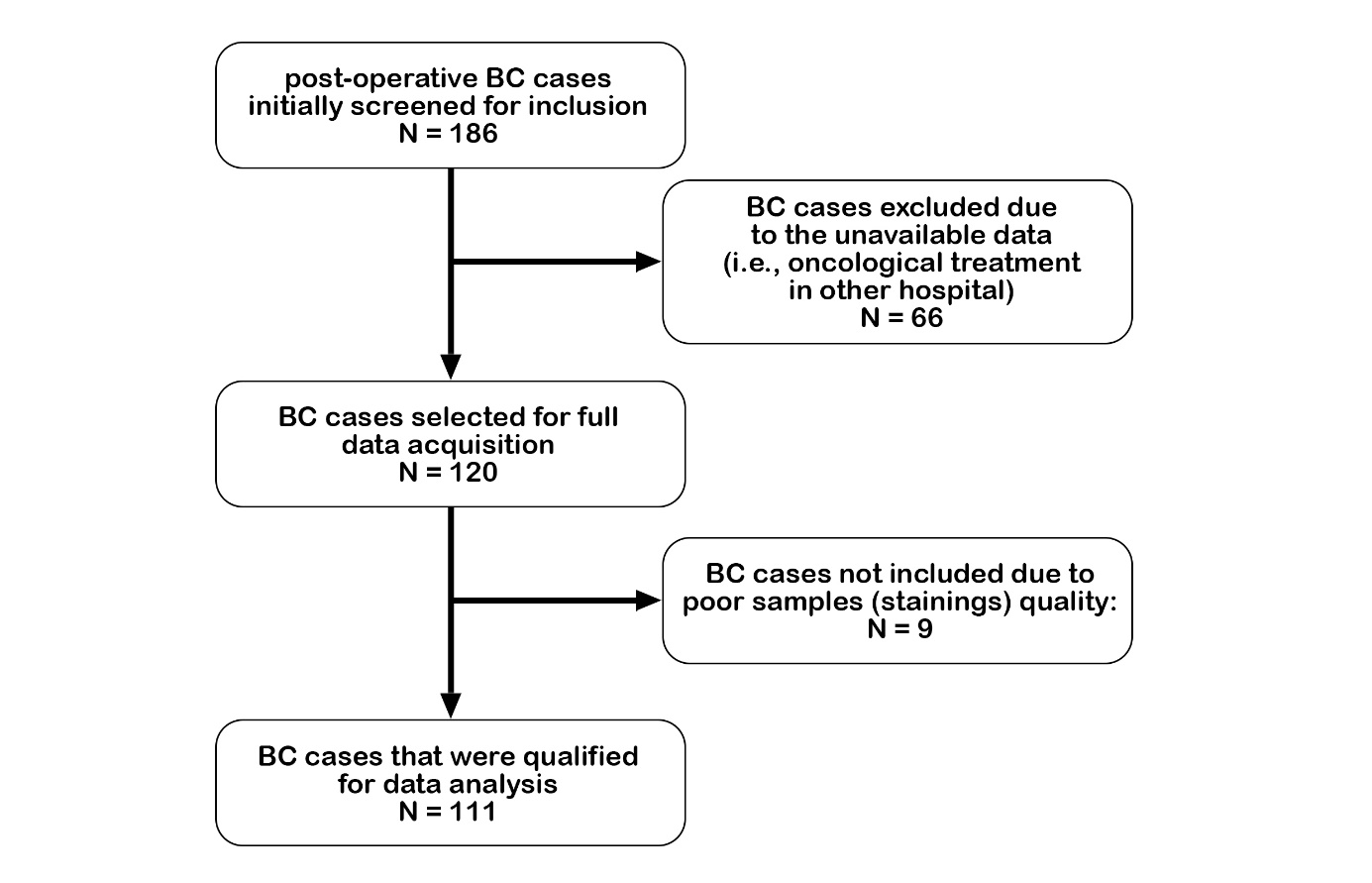


**Abbreviations:** BC—breast cancer.

**
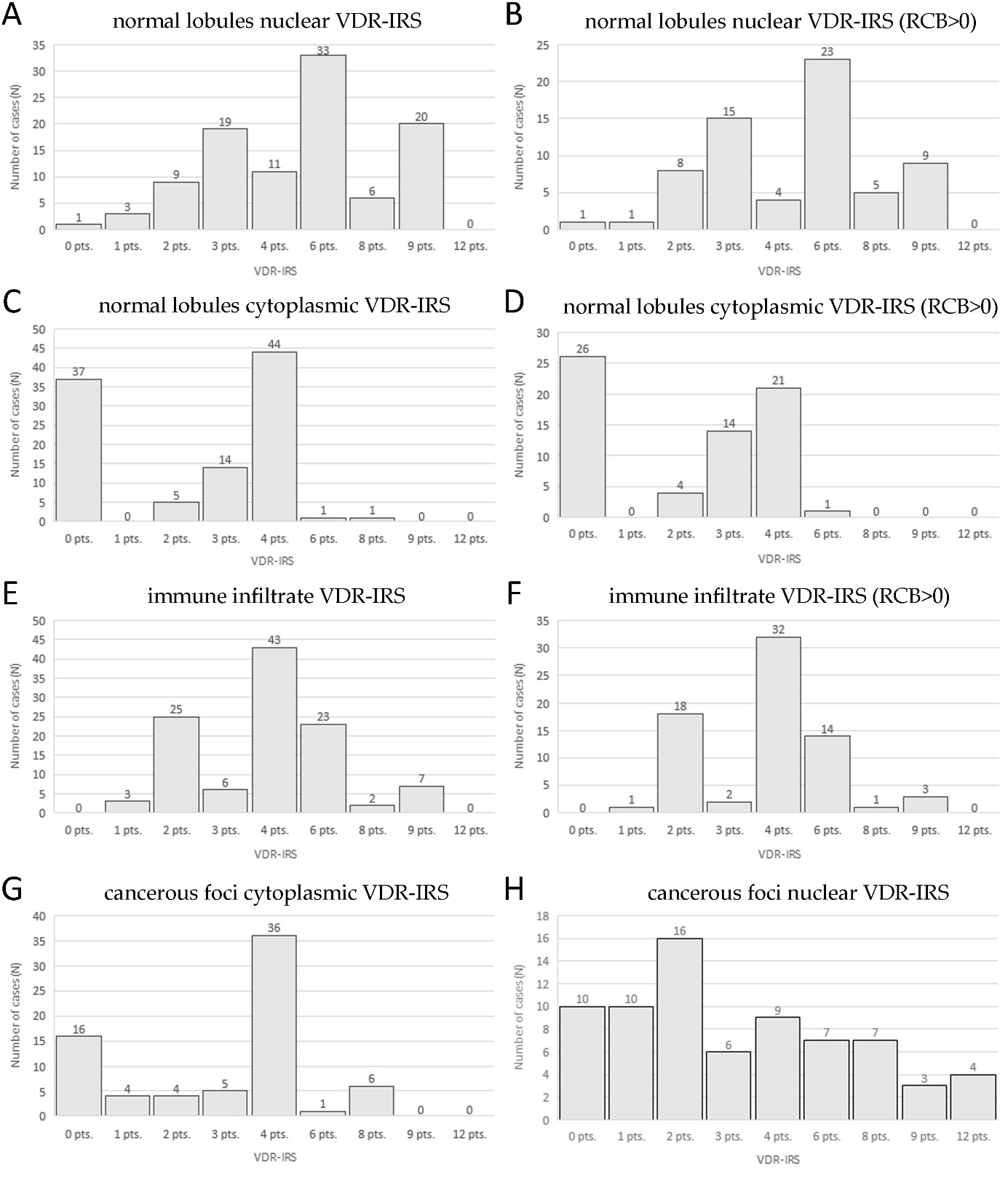
Supplementary Figure S2**. Histograms presenting the frequencies of scores of different VDR-IRS categories. **Abbreviations**: RCB—residual cancer burden; VDR-IRS—vitamin D receptor immunoreactive score.


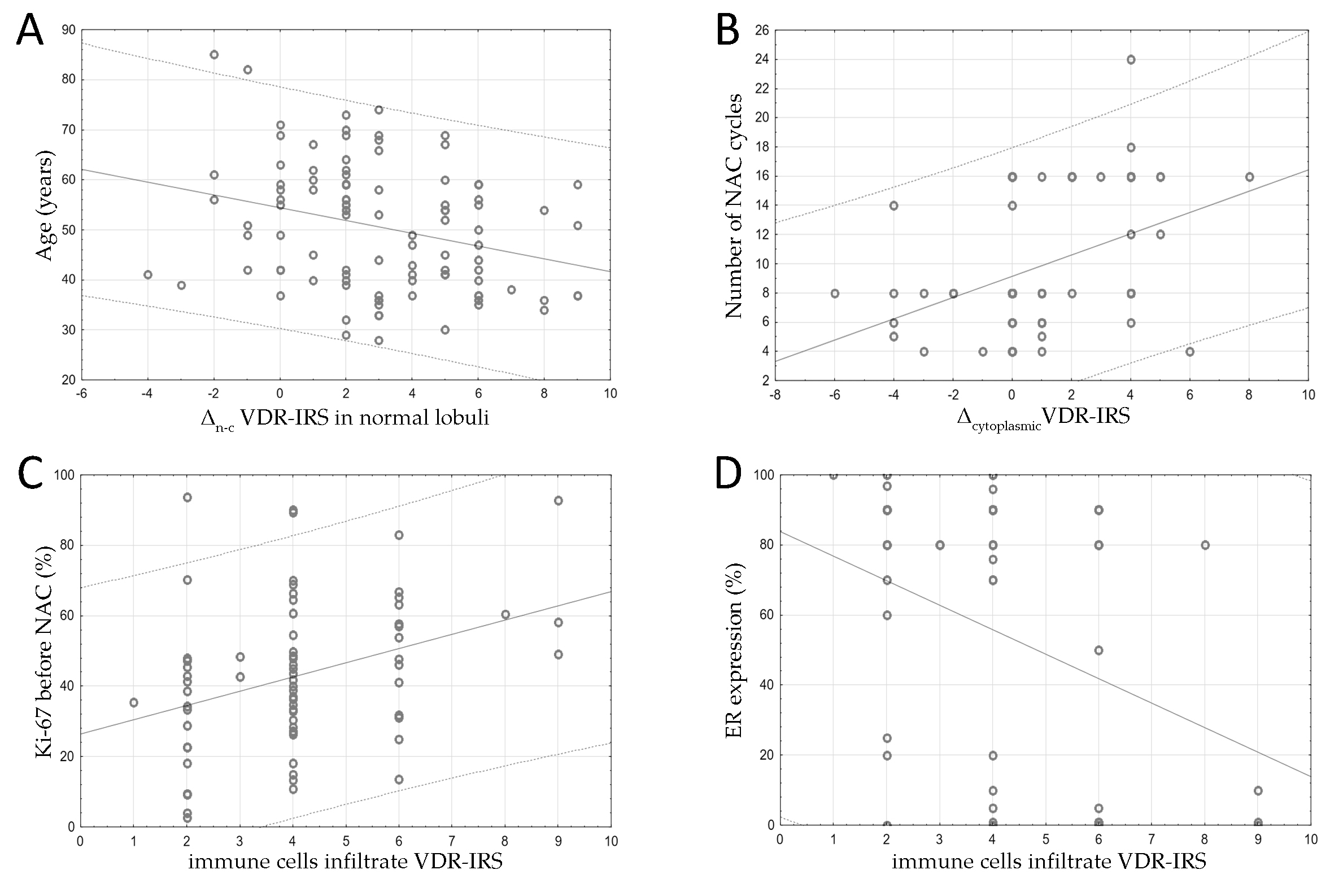


**Supplementary Figure S3**. Correlation diagrams of the relationships that remained statistically significant after application of Benjamini-Hochberg correction. **Abbreviations**: ER—estrogen receptor, NAC—neoadjuvant chemotherapy, RCB—residual cancer burden.
